# Supplementary material for: The Discovery of Small ERK5 Inhibitors via Structure-Based Virtual Screening, Biological Evaluation and MD Simulations
Source: Molecules. 2025 Oct 25;30(21):4181. doi: 10.3390/molecules30214181 (PMC12610425; doi:10.3390/molecules30214181)
Supplement: Supplementary file 1 [file molecules-30-04181-s001.zip › molecules-3877650-supplementary.pdf]

The Discovery of Small ERK5 Inhibitors via Structure-Based Virtual Screening, Biological Evaluation and MD Simulations

Noor Atatreh [1,2], Radwa E. Mahgoub [1,2], Rose Ghemrawi [1,2], Molham Sakkal [1,2], Nour Sammani [1,2], Mostafa Khair [3] and Mohammad A. Ghattas [1,2,*]

1 College of Pharmacy, Al Ain University, Abu Dhabi 64141, United Arab Emirates; noor.atatreh@aau.ac.ae (N.A.); rose.ghemrawi@aau.ac.ae (R.G.)

2 AAU Health and Biomedical Research Centre, Al Ain University, Abu Dhabi 64141, United Arab Emirates

3 Core Technology Platforms, New York University Abu Dhabi, Abu Dhabi 129188, United Arab Emirates; mrk6@nyu.edu

* Correspondence: mohammad.ghattas@aau.ac.ae

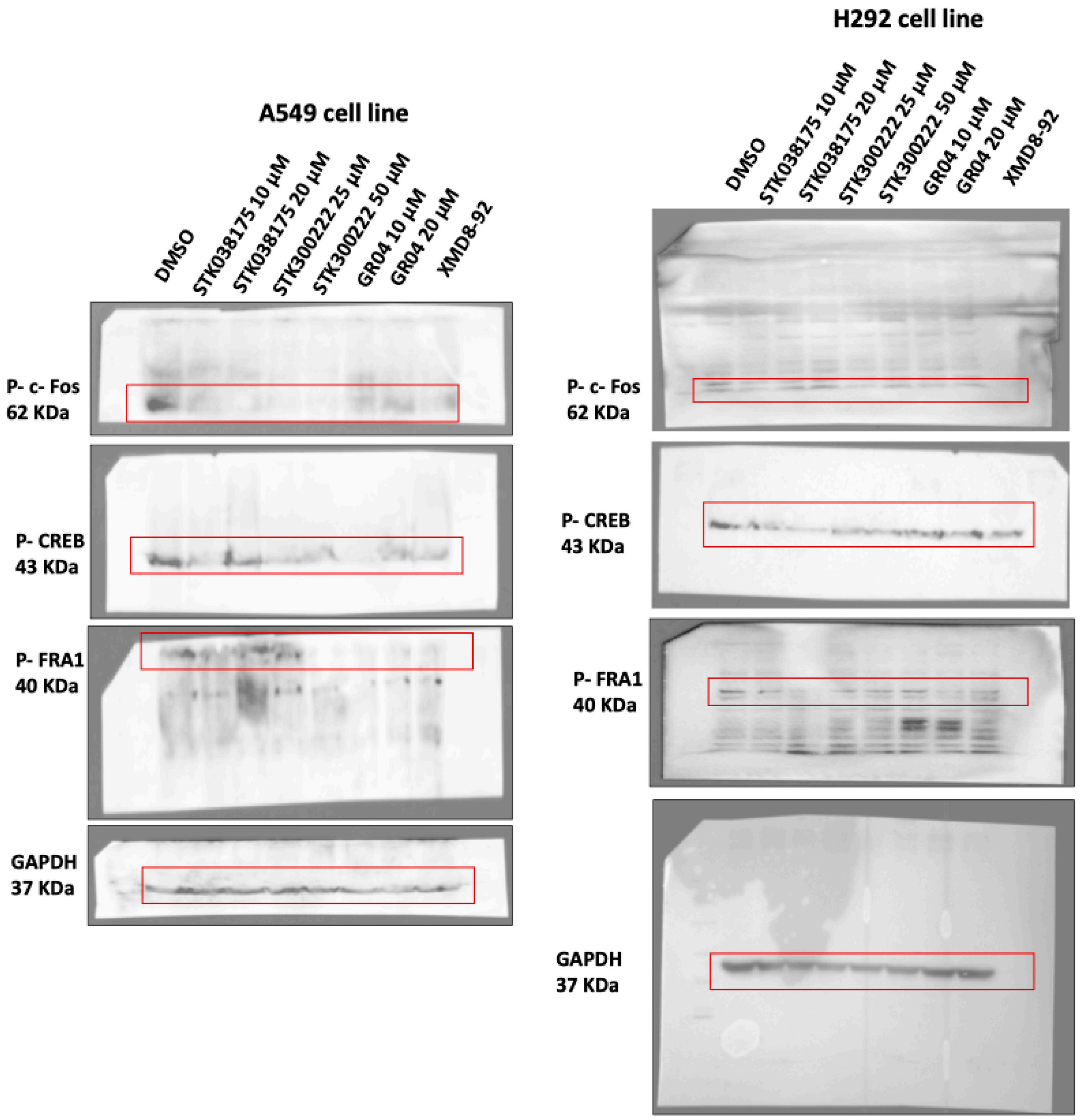

Figure S1. Raw Western blot data for A549 and H292 cell lines. Membranes were cut after blocking to enable the detection of different proteins within the same experiment.

Table S1. Chemical structures of all tested hits.

STK895603	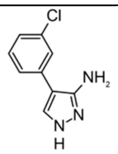	STK346604	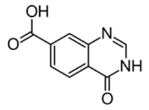
STK781584	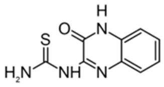	STL357559	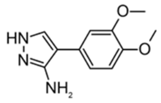

STL308355	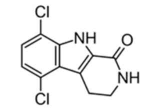	STK311762	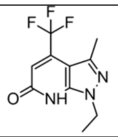
STK688693	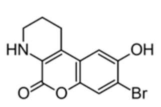	STK795341	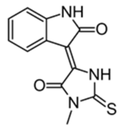
STK695608	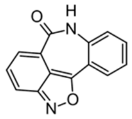	STL373573	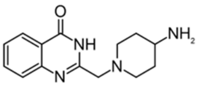
STK189669	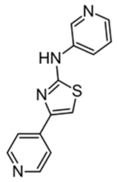	STL310152	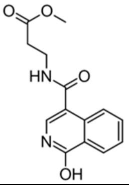
STK025810	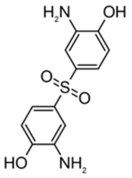	STK421878	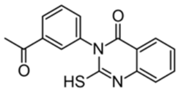
STK309039	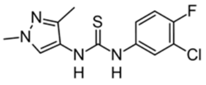	STK300222	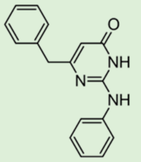
STK038175	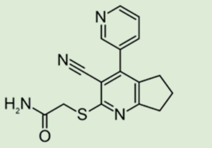	ST096370	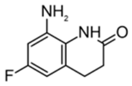
STK682307	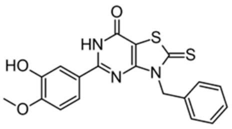	ST075581	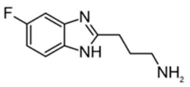
STK305244	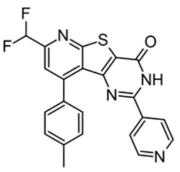	HTS08709	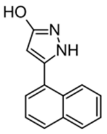
ST044783	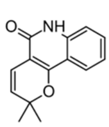	ST101629	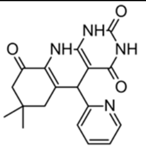

ST50136051	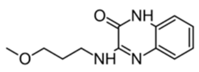	ST013384	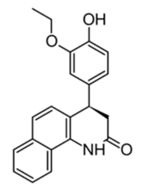
ST075787	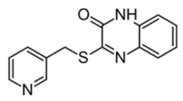	ST064804	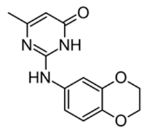
ST078147	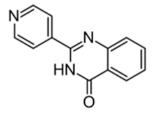	ST060365	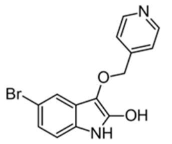
ST053785	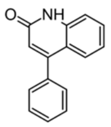	ST50015333	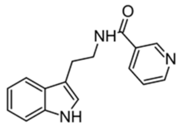
ST071035	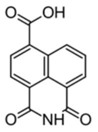	ST085386	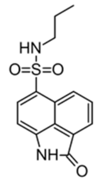
ST4150542	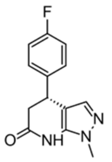	ST073247	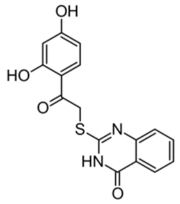
ST50682039	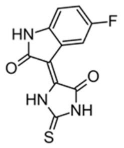	GR04	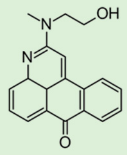
ST085385	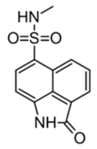	GR04_1	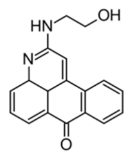

Green highlighted cells: only active compounds

Table S2. MM-GB/SA Scores using different time frames.

Ligand	MM-GB/SA during the first 30 ns (1 – 30 ns) (Kcal mol-1)	MM-GB/SA during the last 30 ns (170 ns – 200 ns) (Kcal mol-1)
5BYZ	-33.78 ± 3.88	-38.96 ± 3.06
GR04	-28.43 ± 3.12	-24.15 ± 3.41
STK038175	-32.97 ± 2.70	-28.76 ± 3.42
STK300222	-23.79 ± 3.31	-35.45 ± 2.71

Table S3. Pairwise assessment raw data for 5BYZ co-crystallized ligand.

5BYZ Co-crystallized Ligand				
	vdW	Electrostatic	Polar Solvation	Non-polar Solvation
Ile61	-3.4664407	0.26789229	-0.360429278	-2.542292092
Tyr66	-1.3756214	-0.20039262	0.048681925	-1.168529322
Val69	-1.7571841	-0.19828118	0.136172476	-1.236444756
Lys84	-0.6969395	-3.92203813	3.038786726	-0.553920782
Glu102	-0.1035684	1.83722654	-1.745999019	-0.024918579
Ile115	-0.6171577	0.02756971	-0.023055203	-0.455130015
Leu137	-0.7858888	-0.10058618	0.153927865	-0.606391376
Asp138	-0.235958	-1.15264706	0.574934372	-0.182259637
Met140	-1.3038718	-3.83777025	0.704809938	-0.986757255
Glu141	-0.8754814	-2.72344464	2.557299046	-0.450287235
Ser142	-1.3490992	-0.47436765	0.074708388	-0.714253991
Asp143	-1.2966225	1.41123797	-1.073364087	-0.762327327
Gln146	-0.70716	-0.01675019	-0.073103538	-0.556122624
Ser186	-0.3758843	-0.31281403	0.258165794	-0.263264139
Asn187	-0.3538218	0.12835582	-0.127533253	-0.207211321
Leu189	-2.2694914	-0.06884569	-0.004586523	-1.70717507
Asp200	-0.5368394	0.08741557	-0.122477059	-0.449590933

Table S4. Pairwise assessment raw data for STK038175 ligand.

STK038175 Ligand				
	vdW	Electrostatic	Polar Solvation	Non-polar Solvation
Ile61	-1.5042682	-0.1326601	0.114022334	-1.205354374
Tyr66	-1.7371764	-0.23810439	0.197237511	-1.377051425
Val69	-2.0433502	-0.0589058	0.069516572	-1.371055051
Lys84	-0.6683768	1.78278719	-1.698169583	-0.549102032
Glu102	-0.1404168	-1.00216544	0.916516926	-0.065023274
Ile115	-0.5078698	-0.20272123	0.186672547	-0.268442388
Leu137	-0.7769234	-0.27573288	0.221584224	-0.572110981
Asp138	-0.2397989	1.42828448	-1.269854501	-0.056768746
Met140	-0.9850713	-2.06806973	0.834277503	-0.761462904
Glu141	-0.5273461	-0.91267937	0.810278524	-0.287032934
Ser142	-0.6319865	-1.45869671	0.268907199	-0.420781185
Asp143	-0.6279621	1.32308012	-1.46755546	-0.336069001
Gln146	-0.0720428	-0.12423326	0.102419963	-0.018917405
Ser186	-0.3786369	-0.04522137	-0.03530157	-0.231854616
Asn187	-0.6840329	-1.49942869	0.135993119	-0.621522633
Leu189	-2.1506262	0.09844829	0.12441087	-1.602603479
Asp200	-0.5862072	-0.31817471	0.11474801	-0.496615042

Table S5. Pairwise assessment raw data for GR04 ligand.

GR04 Ligand				
	vdW	Electrostatic	Polar Solvation	Non-polar Solvation
Ile61	-1.3943334	-0.4778009	0.2845750	-1.2001920
Tyr66	-2.2811954	-0.2588531	0.3697937	-1.7410326
Val69	-1.7852785	-0.2355075	0.1426543	-1.2867252
Lys84	-0.8903214	-4.2157320	3.2478379	-0.7413243
Glu102	-0.0803000	1.4257136	-1.3931214	-0.0209378
Ile115	-0.5641964	-0.0104487	0.0025372	-0.4119867
Leu137	-0.7544487	0.0449843	0.0223227	-0.6844588
Asp138	-0.2180966	-1.4831243	1.2072520	-0.1657824
Met140	-1.4009193	-0.4898074	0.3481993	-1.0118493
Glu141	-0.4442637	-1.7059783	1.7007603	-0.2762908
Ser142	-0.4105213	-0.1815874	0.0647333	-0.2138948
Asp143	-0.6043370	-1.9980578	1.5089558	-0.5160297
Gln146	-0.1429955	0.1690633	-0.2091888	-0.0842202
Ser186	-0.4217520	0.1754065	-0.2000887	-0.3011559
Asn187	-0.3507986	-0.0502179	-0.0430687	-0.1769575
Leu189	-2.1133129	-0.0031718	0.0099127	-1.5859091
Asp200	-0.4301966	0.3290682	-0.3206076	-0.3262858

Table S6. Pairwise assessment raw data for STK300222 ligand.

STK300222 Ligand				
	vdW	Electrostatic	Polar Solvation	Non-polar Solvation
Ile61	-0.4370145	-0.22420395	0.139671035	-0.396717395
Tyr66	-3.012129	-1.16724446	0.046295189	-2.231434618
Val69	-1.0505948	-0.06057093	0.056843713	-0.826812866
Lys84	-0.7690689	-7.19623764	4.078395661	-1.010646875
Glu102	-0.2852877	0.90718094	-0.834150472	-0.154428459
Ile115	-0.7902893	0.09678465	-0.093274313	-0.715782192
Leu137	-0.8238931	0.11088055	-0.086741606	-0.822919476
Asp138	-0.2116683	-0.66924244	0.621234969	-0.131680675
Met140	-0.5907553	-0.17247481	0.119756214	-0.407472097
Glu141	-0.0666819	-0.32857792	0.344054982	-0.017124295
Ser142	-0.0761812	0.05969345	-0.054599028	-0.019115554
Asp143	-0.1933984	-0.67161581	0.633833076	-0.055724669
Gln146	-0.0203309	-0.00210273	0.002753427	-0.000698595
Ser186	-0.959381	-0.98952468	0.145482103	-0.777966117
Asn187	-1.2419567	-0.4953884	0.047533384	-0.641014481
Leu189	-1.4455863	-0.1202348	0.082338821	-1.282630068

Asp200	-1.6902141	0.2352662	-0.115551301	-1.12421603
